# Potential Antioxidant and Anti-Inflammatory Properties of Serum from Healthy Adolescents with Optimal Mediterranean Diet Adherence: Findings from DIMENU Cross-Sectional Study

**DOI:** 10.3390/antiox10081172

**Published:** 2021-07-23

**Authors:** Giuseppina Augimeri, Angelo Galluccio, Giovanna Caparello, Ennio Avolio, Daniele La Russa, Daniela De Rose, Catia Morelli, Ines Barone, Stefania Catalano, Sebastiano Andò, Cinzia Giordano, Diego Sisci, Daniela Bonofiglio

**Affiliations:** 1 Department of Pharmacy, Health and Nutritional Sciences, University of Calabria, 87036 Rende (CS), Italy; giusy.augimeri@gmail.com (G.A.); daniele.larussa@unical.it (D.L.R.); daniela.derose89@gmail.com (D.D.R.); catia.morelli@unical.it (C.M.); ines.barone@unical.it (I.B.); stefcatalano@libero.it (S.C.); sebastiano.ando@unical.it (S.A.); cinzia.giordano@unical.it (C.G.); diego.sisci@unical.it (D.S.); 2 Health Center Srl, 87100 Cosenza, Italy; angelo.galluccio@yahoo.it (A.G.); caparello.giovanna@gmail.com (G.C.); ennioavolio@libero.it (E.A.); 3 Department of Clinical and Experimental Medicine, University Magna Graecia, 88100 Catanzaro, Italy; 4 School of Specialization in Food Sciences, University of Rome Tor Vergata, 00133 Rome, Italy; 5 Centro Sanitario, University of Calabria, 87036 Rende (CS), Italy

**Keywords:** Mediterranean Diet, antioxidant capacity, anti-inflammatory effects, cytokines, oxidative stress, adolescents, polyunsaturated fatty acids, C-Reactive Protein, Erythrocyte Sedimentation Rate, human macrophages

## Abstract

During adolescence, health status is influenced by several factors, among which dietary pattern is a crucial element of lifestyle in terms of prevention and treatment of metabolic and chronic diseases. The most studied healthy dietary pattern is the Mediterranean Diet (MD), due to a combination of foods that are rich in antioxidant and anti-inflammatory nutrients. The aim of this study, carried out in healthy adolescents from the DIMENU study, is to assess the adherence to the MD, as well as the dietary nutrient intake and to evaluate the potential antioxidant and anti-inflammatory properties of sera from participants grouped according to the MD score. Using the KIDMED score, as the MD quality index for children and teenagers, we found that the adolescents in this study had an average adherence to the MD (6.71 ± 2.58). Adolescents were clustered into three groups based on their MD adherence. Assessment of quality by 24 h recall revealed higher intakes in polyunsaturated fatty acid (PUFA)/saturated fatty acid (SFA) ratio, dietary fibers, vitamins, and total oxygen radical absorbance capacity (ORAC) in the optimal than in poor MD adherence group. We observed that dietary PUFA/SFA ratio was negatively correlated with serum C-Reactive Protein levels, and total dietary fibers were inversely correlated with Erythrocyte Sedimentation Rate values, while total ORAC was directly correlated with serum glucose concentrations. Interestingly, the reactive oxygen metabolite (ROM) concentrations, determined by the ROM assay, were significantly lower in pooled sera from optimal than poor adherers. Finally, using lipopolysaccharide-stimulated human macrophages, as an in vitro model of acute inflammation, we found a reduced secretion of pro-inflammatory cytokines upon serum treatment from adolescents with optimal respect to medium and poor MD adherence. Our results highlight the anti-inflammatory and antioxidant properties of serum from adolescents with healthy nutrition in terms of adherence to the MD, which may have a positive impact on the prevention of chronic diseases in adulthood.

## 1. Introduction

The Mediterranean Diet (MD) is considered one of the healthiest dietary patterns, due to a combination of foods, rich in antioxidants and anti-inflammatory nutrients, which have been proven to exert a positive role against several metabolic and chronic degenerative diseases [1,2,3,4]. The MD pattern consists of the high consumption of fruits, vegetables, whole grains, legumes, nuts, olive oil, and low fat dairy products, in moderate consumption of fish, and in limited intake of meat [5,6]. The basic principles of MD refer to the quality of diet defined by a food-based pattern, which appears to have a greater effect on health outcomes compared with a nutrient-based approach. Particularly, the quality of the MD pattern is characterized by regular consumption of nutrients, such as monounsaturated fatty acids (MUFAs) and polyunsaturated fatty acids (PUFAs), high consumption of polyphenols, fibers, and low glycemic carbohydrates, and greater intake of plant proteins than animal proteins [7,8]. Conversely, the low intake of saturated fat (SFA) in the MD is related to the low consumption of meat, fat milk, and butter, despite a relatively high intake of total fat coming predominantly from extra-virgin olive oil, a wide variety of nuts, seeds, and the germ of whole grains. Nuts, in particular almonds, walnuts, hazelnuts, and peanuts, are a very good source of PUFAs and plant sterols, which are anti-inflammatory compounds [4,9]. Overall, the low amounts of SFA and the high consumption of PUFAs and micronutrients, including dietary vitamins and minerals, are commonly reported for empowering of the plasma antioxidant capacity and anti-inflammatory properties leading to benefits for cardiovascular disease, bone health, gastrointestinal and cognitive functions [4,10,11,12,13].

Several factors, including dietary patterns and physical activity, influence health status over adolescence. These lifestyle elements are crucial in both the prevention and treatment of metabolic and chronic diseases [14,15,16]. We investigated the impact of the adherence to the MD and different levels of physical activity on metabolic parameters, in adolescents from a Mediterranean area, evaluating potential predictors of health status in adolescents [17]. Moreover, the promotion of healthy food consumption should be fostered by a specific nutritional educational approach for a conscious food choice [18]. Recently, during the coronavirus disease 2019 (COVID-19) pandemic, we assessed the eating behavior, including the intake of Mediterranean foods in Italian adolescents, and we found no substantial differences in dietary habits with respect to pre-lockdown period in terms of the adherence to the MD and their food choices, even if active adolescents were higher adherent respect to sedentary ones [19,20,21,22]. However, since adolescents are in a vulnerable age group, good nutrition, and a healthy diet are essential to maintain optimal health, boost their immune system and prevent communicable and non-communicable diseases. Thus, dietary assessments, including questionnaires and dietary recalls, to evaluate the MD adherence and to estimate individual nutrient intakes, are commonly used as cost-effective research tools in healthcare practice and clinical research studies [23,24]. The aim of this investigation, carried out in healthy adolescents living in Southern Italy in the context of DIMENU study after the lockdown period of COVID-19 pandemic, is to assess the adherence to the MD, as well as the dietary nutrient intake by 24 h recall and to evaluate the potential antioxidant and anti-inflammatory properties of adolescents’ sera grouped according to the MD score.

## 2. Materials and Methods

### 2.1. Study Population

The DIMENU (Dieta Mediterranea and Nuoto) project was funded by the EU Regional Operational Program Calabria, Italy (prot. #52243/2017) for investigating the impact of the adherence of the MD and physical activity on healt status in a sample of adolescents from Southern Italy as cross-sectional [17] and longitudinal [25] studies. As part of the DIMENU project, in the current investigation, we recruited a total of 77 subjects, including 36 girls and 41 boys, aged 14 to 17 years, from public high school students. The exclusion criteria were cognitive or physical/motor limitation, health-related problems, use of medications, and a restrictive diet. A detailed explanation of study purposes was provided to all participants and their parents, which gave written informed consent, prior to their enrollment in the DIMENU study. The study was carried out in accordance with the guidelines laid down in the Declaration of Helsinki and approved by the Ethics Committee of the University of Calabria, Italy (#5727/2018).

### 2.2. Anthropometric Parameters and Bioelectrical Impedance Analysis

A detailed description of the anthropometric measurements and bioimpedentiometric analysis (BIA) performed has been reported elsewhere [17]. BIA estimated phase angle (PhA), fat-free mass (FFM), and fat mass (FM), total body water (TBW), body cell mass (BCM). Data obtained by BIA test were analyzed using Version 1.2.2.8. of the software Bodygram Plus (Akern Srl; Florence, Italy).

### 2.3. KIDMED Score

The Mediterranean Diet Quality Index for children and teenagers (KIDMED test) was used to assess adherence to the MD. The score of MD adherence was based on a 16-point paper questionnaire in which a value of +1 was assigned for the intake of whole cereals or grain, vegetables, fruits, legumes, dairy products, fish, nuts, yogurt, olive oil, and a negative value −1 for skipping breakfast and eating fast food, as well as for consumption of baked goods and sweets. Thus, twelve questions are positively scored, and four are negatively scored. Based on the KIDMED scores, which range from 0 to 12, adolescents were classified as follows: optimal (≥8 points), medium (4–7 points), and poor (≤3 points) adherence to the MD [26].

### 2.4. Dietary Assessment by 24 h Recall

Subjects provided information on their daily meals through a 24 h recall completed via an interview conducted by nutritionists. The dietary intake assessment was very accurate, since data were collected in an in-depth interview manner which required 20 to 30 min each subject to complete a single day recall. Moreover, detailed data about food preparation methods, ingredients used in mixed dishes, and the estimation of the amounts of each food consumed in reference to a common size container (e.g., bowls, cups, and glasses), standard measuring cups, and spoons were collected using a photographic atlas of food portions designed as a tool to visually estimate food amounts (Supplementary Figure S1). Nutrient intakes were calculated by multiplying the portion weight by its nutrient content. Specific software MetaDieta software Vers. 4.2.1. (Meteda S.r.l, Roma, Italy) which includes the Italian database, was used to analyze the energy and nutrient content of food intake.

### 2.5. Biochemical and Hormonal Measurements

Fasting blood samples were centrifuged, and serum biochemical parameters were analyzed, as previously described [25]. Erythrocyte Sedimentation Rate (ESR) was measured by the Wintrobe method. Serum C-Reactive Protein (CRP) levels were detected by immunonephelometry (GOLDSITE Diagnostics, Inc., Shenzhen, China). Serum insulin levels were determined with an Enzyme-Linked Immunosorbent Assay (ELISA) kit (Novatec Immundiagnostica GmbH, Dietzenbach, Germany) following the manufacturer’s instructions [17]. Insulin action was expressed as Homeostasis Model Assessment for estimating Insulin Resistance (HOMA-IR) which was calculated as the product of fasting glucose concentration (mg/dL) and fasting insulin concentration divided by 405.

### 2.6. Cell Culture and Experimental Treatments

A human THP-1 monocytic cell line was acquired from American Type Culture Collection (ATCC, Manassas, VA, USA), authenticated, and stored according to the supplier’s instructions. For experiments, 500,000 THP-1 cells were seeded in 12 multi-well dishes and differentiated in M0 macrophages with phorbol 12-myristate 12-acetatate 100 nM (PMA, Sigma-Aldrich, Schnelldorf, Germany) for 24 h followed by 1 day of rest [27]. To study the effects of serum from adolescents, M0 macrophages were polarized in M1 macrophages by stimulation with 10 ng/mL lipopolysaccharide (LPS, Sigma-Aldrich, Schnelldorf, Germany) in serum free medium for 6 h. Subsequently, the medium was supplemented with 10% of pooled sera of adolescents stratified with respect to MD adherence.

### 2.7. Cytokine Measurement

Culture supernatants were collected from M1 macrophages cultured in the presence of serum from adolescents after 24 h. IL-6 and TNF-α levels were measured using the ELISA kit (Sigma-Aldrich, Schnelldorf, Germany) according to the manufacturer’s instructions. Results are presented as pg/mL.

### 2.8. ROM and BAP Assays

Reactive Oxygen Metabolites (ROM) and Biological Antioxidant Potential (BAP) determination were performed by using photometric measurement kits and a free radical analyzer system provided with spectrophotometric device reader (FREE Carpe Diem, Diacron International, Grosseto, Italy) [28,29]. The d-ROM test helps to determine the oxidant ability of a plasma/serum sample by measuring the presence of reactive oxygen metabolite derivatives, in particular, hydroperoxides (oxidative index). Results are expressed in Carratelli units (UC; 1UC = 0 8 mg/L of hydrogen peroxide). The BAP test measures the blood concentration of antioxidants capable of reducing the iron from ferric to the ferrous form (antioxidant barrier). Results are expressed in μmol/L of the reduced ferric ions.

### 2.9. Statistical Analysis

Data were reported as the mean and standard deviation (SD), and statistical differences between samples were evaluated by using parametric tests (one-way ANOVA and Student’s *t*-test). Qualitative variables were reported as frequencies (%), and the statistical differences were evaluated by Chi-squared tests. The correlation between variables was evaluated by Spearman’s correlation test. Statistical significance was set at *p* < 0.05.

## 3. Results

### 3.1. Characteristics of Participants and Adherence to the Mediterranean Diet

The general characteristics of the total study population and differentiated by sex are shown in Table 1. The mean age of the total population studied was 15.77 (±1.07) years without gender differences. Normal mean values of BMI were found in the total adolescent sample (22.87 ± 3.39), showing a proportion of participants with overweight and obesity of 24.67% and 3.89%, respectively, with overweight and obesity percentages being greater in boys than in girls (15.58% vs. 9.09%, and 2.59% vs. 1.3%, respectively). We also reported data on waist/hip ratio along with BCM, FM, FFM, PhA, and TBW, which reflect an indirect measure for body composition. In addition, we measured indicators of glycemic (glucose, insulin, and HOMA-IR) and lipid (triglycerides, Cholesterol, LDL, HDL) profile, kidney (creatinine and urea nitrogen) and liver (total and direct bilirubin) function, and serum uric acid concentrations, which resulted at normal values. Similarly, we found normal concentrations of ESR and CRP levels. Using the KIDMED score, we evaluated the MD adherence, which was 6.71 (±2.58) for the total sample independently of sex, indicating an average adherence to the MD (Table 1).

Based on the KIDMED values, all adolescents were divided into optimal (score ≥ 8), medium (score 4–7) and poor (score ≤ 3), adherence to the MD [26], and the percentage of participants who had medium adherence were around 50%, more than 40% of adolescents declared an optimal adherence, whereas less than 10% had poor adherence to the MD (Table 2).

**Figure 1:**
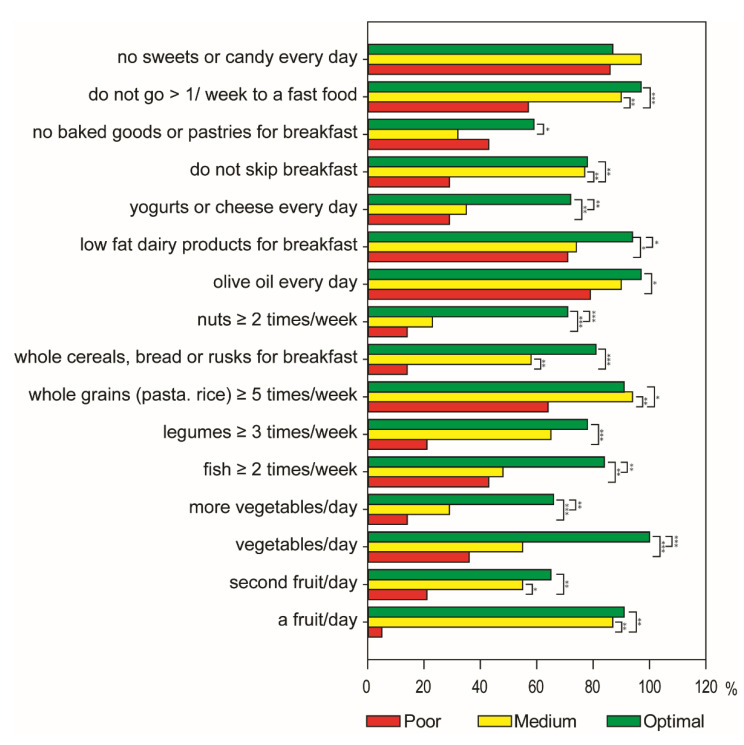
Compliance with items from the KIDMED test according to the poor, medium, and optimal adherence to the Mediterranean diet in the total sample. The percentage (%) of the population adherent to each recommendation is reported in the bar chart. Statistical differences were evaluated by Chi-square test (* *p* ≤ 0.05, ** *p* ≤ 0.01, *** *p* ≤ 0.001).

Particularly, the differences in the compliance rates for each food were calculated according to the three subgroups and reported in Figure 1. As expected, significant differences for most of the items were observed between optimal and poor adherence to the MD group, except for ‘second fruit/day’, ‘olive oil every day’, ‘low fat dairy products for breakfast’, ‘no baked goods or pastries for breakfast’ and ‘no sweets or candy every day’, which is almost within the recommendations among the three classes of MD adherence. In addition, the intakes of ‘vegetables/day’, ‘fish ≥2 times/week’, ‘low fat dairy products for breakfast’, and ‘yogurts or cheese every day’ were significantly higher in optimal than in medium MD adherence group. Conversely, adolescents with poor adherence to the MD showed most of the items outside the recommendations and only the consumption of ‘whole grains (pasta, rice) ≥5 times a week’, ‘olive oil every day’ and ‘low fat dairy products for breakfast’ was 57%, 86%, and 71%, respectively, within recommendations according to the KIDMED score (Figure 1). Particularly, the intakes of ‘more vegetables a day’, ‘legumes ≥3 times a week’, ‘whole cereals, bread or rusks for breakfast’, ‘nuts ≥ two times a week’ and ‘yogurts or cheese every day’ were above 25% of the recommendation (Figure 1).

### 3.2. Dietary Intake Assessment by a 24 h Recall in Adolescents

With increased focus on assessing the dietary intake among adolescents, subjects were invited to recall, through a face-to-face interview with nutritionists, details of foods and drinks consumed over the previous 24 h (Supplementary Table S1).

**Figure 2:**
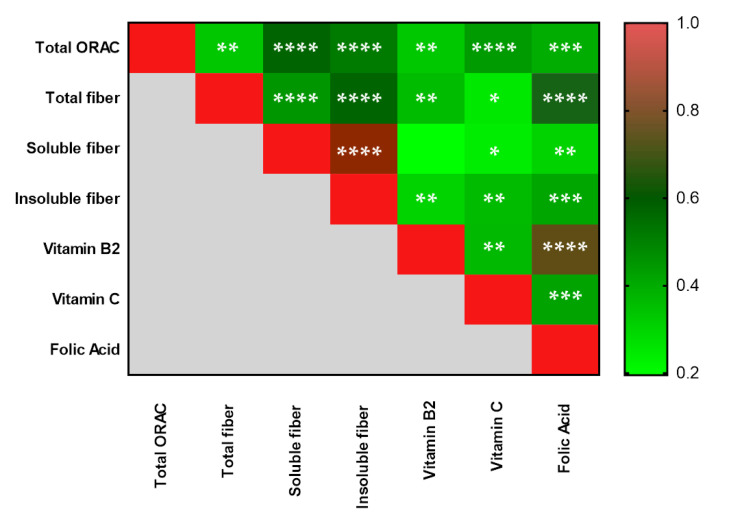
Heatmap representing significant positive correlations among nutrients measured in the whole cohort. ORAC, Oxygen Radical Absorbance Capacity. The correlation coefficient range (0–1) is color-coded (green to red). Correlations were made using Spearman’s correlation analysis (* *p* ≤ 0.05, ** *p* ≤ 0.01, *** *p* ≤ 0.001, **** *p* ≤ 0.0001).

Using a Spearman’s correlation analysis, we investigated the potential correlation among intakes of nutrients. Interestingly, there were significant positive correlations among total ORAC, total fiber, soluble and insoluble fiber, vitamin B2, Vitamin C, and folic acid (Figure 2).

In addition, we evaluated the correlations between dietary nutrients and body composition parameters, as well as general metabolic, health, and inflammatory biomarkers in all adolescents. We found that total ORAC was inversely related to serum glucose concentration (*r* = −0.239, *p* = 0.038), while total dietary fibers were positively correlated with FFM (*r* = 0.248, *p* = 0.031) and inversely related to FM (*r* = −0.314, *p* = 0.005). Interestingly, total dietary fibers were inversely correlated with ESR values (*r* = −0.312, *p* = 0.005), while PUFA/SFA ratio was negatively correlated with serum CRP levels (*r* = −0.262, *p* = 0.021).

Thus, we analyzed the differences in the total energy intake and nutrients among adolescents grouped according to the poor, medium, and optimal adherence to the MD evaluated by the KDMED score (Table 3). Interestingly, we observed that, even in the presence of a similar total energy intake among the three groups, optimal adherers showed significant higher intakes in PUFA/SFA ratio, total dietary fiber, soluble and insoluble dietary fiber, vitamins B2, C, and total oxygen radical absorbance capacity (ORAC) values than subjects with poor adherence to the MD. Both adolescents with medium and optimal MD adherence had a higher intake of soluble sugars along with a significantly reduced glycemic load than those with poor MD adherence (Table 3).

According to the categorization of our adolescents into the three MD adherence groups, we analyzed anthropometric and body composition parameters, as well as metabolic indicators of glycemic and lipid profile, kidney and liver biochemical markers, and serum uric acid concentrations. In the statistical analysis of variance, we did not observe any significant variation among the three different groups (Supplementary Table S1). We also evaluated the inflammatory status by measuring ESR and CRP, which are the most common biomarkers widely used for identifying and monitoring individuals with systemic inflammatory activity. Unsurprisingly, in our healthy adolescents, no differences were observed in these inflammatory biomarkers (Supplementary Table S1).

### 3.3. Antioxidative and Anti-Inflammatory Properties of Serum Samples from Adolescents Classified According to the Three Mediterranean Diet Adherence Groups

**Figure 3:**
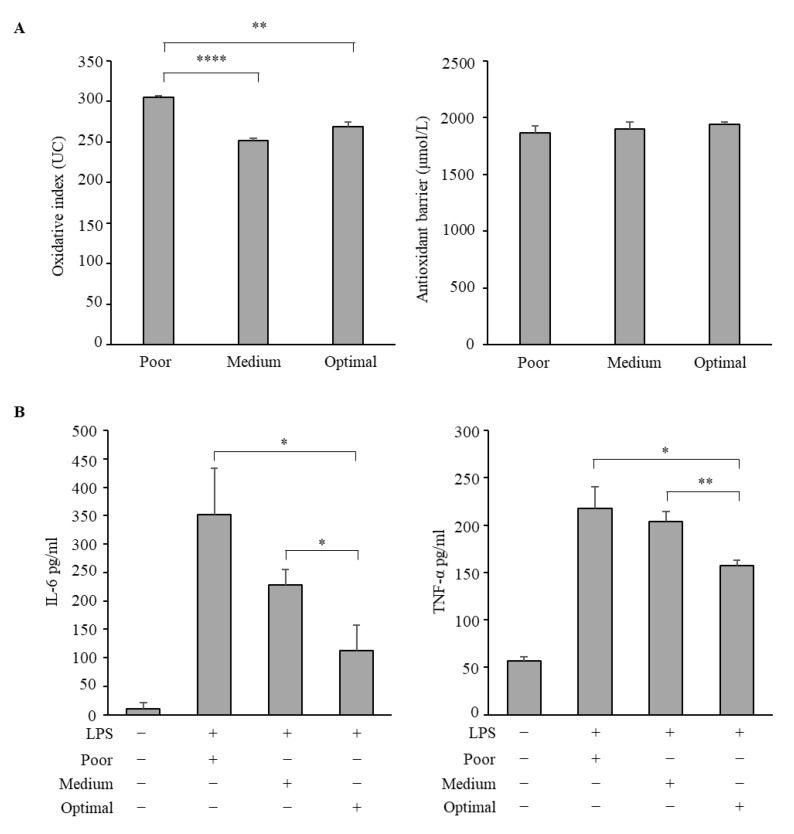
Antioxidant and anti-inflammatory effects of sera from adolescents stratified with respect to the Mediterranean Diet adherence. (**A**) d-ROM (left panel) and BAP (right panel) assay in adolescent sera. (**B**) THP-1 derived macrophages (M0 macrophages) were subjected to 10 ng/mL lipopolysaccharide (LPS) stimulation for 4 h (M1 macrophages), followed by 24 h incubation with a pool of serum (10% *v/v*) from adolescents stratified with respect to the Mediterranean Diet adherence. Enzyme linked immunosorbent assay (ELISA) for IL-6 (left panel) and TNF-α (right panel) protein secretion in M0 macrophages (−) and M1 macrophages incubated with sera from adolescents with poor, medium, or optimal adherence to the MD. M1 macrophages incubated with a pool of sera from all adolescents were used as control. The values represent the mean ± SEM of three different experiments, each performed in duplicate. * *p* < 0.05; ** *p* < 0.01; **** *p* < 0.0001.

Based on the significantly higher intakes of nutrients rich in vitamins, PUFAs, and fibers in optimal than in subjects with poor adherence to the MD, we considered exploring the potential antioxidant and anti-inflammatory properties of serum samples. Thus, we first determined the concentration of both lipid peroxidation products and total antioxidant status of serum from the three groups of adolescents with optimal, medium, and poor MD adherence. Interestingly, we observed that the hydroperoxide concentrations, determined by the d-ROM assay, were significantly higher in serum samples from poor than in medium, as well as optimal adherers, while no differences were found in serum antioxidant levels, measured by the BAP assay (Figure 3A).

Then, we used LPS-stimulated human monocytes/macrophages, as an in vitro model of acute inflammation, to explore the anti-inflammatory activities of polled sera from adolescents. Specifically, we evaluated by ELISA the secretion of IL-6 and TNF-α in activated macrophages after treatment with serum from poor, medium, and optimal adherers. As shown in Figure 3B, untreated macrophages, named M0 macrophages, secrete IL-6 and TNF-α at a very low level, while the treatment of LPS-stimulated macrophages with serum from optimal adherers significantly reduced the secretion of both IL-6 and TNF-α in comparison with the other two polled sera, indicating the ability of serum of adolescents who had optimal MD adherence to decrease the pro-inflammatory cytokine production in LPS-stimulated macrophages.

## 4. Discussion

In this cross-sectional study, we evidenced the antioxidant and anti-inflammatory properties of serum from adolescents who declared an optimal adherence to the MD, reinforcing the healthy benefits of the Mediterranean-style diet pattern. We firstly assessed in our population the MD adherence using the KIDMED test, and we identified an average adherence to the MD with a mean KIDMED score of 6.71 ± 2.58 for the total sample irrespective of sex. In particular, when analyzing the results of the KIDMED test by the three adherence groups, we found that 48% of participants were medium adherers, 43% had optimal adherence to the MD, and only 9% were poor adherers. These data showed better compliance with the recommendations of the MD with respect to our previous investigation in which poor 16.3%, medium 60.87%, and optimal adherence 22.83% in adolescents were observed [17]. Other authors have reported a medium adherence to the MD in adolescents living in the Mediterranean area and in countries outside the Mediterranean basin [30,31,32]. Recently, in the same geographical region, we found medium adherence to the MD in the adult population even though moving away from the traditional patterns has been identified in younger people [33]. Referring to the compliance with items from the KIDMED test in our adolescent sample, according to the MD adherence, the frequency for consuming fruit, vegetables, fish, legumes, whole grains, whole cereals for breakfast, nuts, olive oil, low fat dairy products, yogurts or cheese every day resulted significantly higher in optimal with respect to poor adherers. Similarly, the results of dietary habits, such as skipping breakfast or eating fast food, were higher in poor than in optimal adherers, indicating better compliance to the healthier behavior in this latter group of adolescents. Regarding the estimation of dietary quality, although the food frequency questionnaire (FFQ) is easy to administer and able to evaluate the diet over an extended period, this method is often self-administered and less precise, particularly for young participants compared with other dietary assessment methods, such as 24 h recalls, which requires a professionally trained interviewer to capture the intra-person variability of diet. For these reasons, 24 h recall is often preferred as the “gold standard” against which an FFQ is calibrated [34,35]. Thus, as the most suitable method to obtain a reliable dietary intake assessment, the detailed 24 h recall was performed by nutritionists to estimate the nutrients intake of our adolescents. Interestingly, PUFA/SFA ratio, total, as well as soluble and insoluble dietary fibers, were significantly elevated in adolescents compliant with the recommendations of the MD with respect to poor adherers, while glycemic index from nutrients was reduced in subjects with optimal respect to those with poor MD adherence. Moreover, in the body composition parameters of adolescents, total dietary fibers were positively correlated with lean mass and negatively correlated with adipose mass. Most scientific evidence showed MD as a model of healthy eating, due to a combination of highly complex carbohydrates in fiber (present in legumes, vegetables, fruits, and cereals), PUFAs with anti-inflammatory activities (found in olive oil and nuts), and bioactive compounds with antioxidative properties, such as flavonoids, phytosterols, terpenes, and polyphenols [36,37,38]. Similarly, a perfect equilibrium of micronutrients, including vitamins and minerals, plays a role in fighting and preventing malnutrition and immunodeficiencies [39]. Regarding micronutrient intake, we found that vitamin C and folic acids, which are widely present in fruits and vegetables, are significantly higher in medium and optimal than in adolescents with poor MD adherence, highlighting these nutrients as reliable markers for fruit and vegetable intake. Mielgo-Ayuso et al. reported that higher consumption of fruit and vegetables is associated not only with increased vitamin intake, but also with plasma vitamin concentrations in adolescents compared with very low consumers [40]. Nutrient-rich foods exert anti-inflammatory action, contributing to attenuating risk factors for chronic degenerative diseases [41]. In fact, inflammation leads to oxidative stress, which, in turn, damages various macromolecules, such as DNA, lipids, and proteins, contributing to the onset of several chronic diseases [42]. Thus, reducing inflammation and oxidative stress may provide chances for the prevention and potential treatment of metabolic and chronic diseases. Although, as expected, we did not find any differences among the anthropometric, body composition parameters and biochemical metabolic index among healthy adolescents, we explored the antioxidant and anti-inflammatory properties of serum from participants categorized according to their adherence to the MD. Based on the serum detection of reactive oxygen metabolites and of biomarkers for the antioxidant status, enabling simultaneous assessment of oxidation degree and antioxidant capacity, we displayed reduced oxidative stress in adolescents with optimal Mediterranean dietary patterns. In the clinical setting, the measurement of d-ROMs and BAP has been reported as valuable biomarkers in children [43]. We also used LPS-stimulated human monocytes/macrophages, which were adopted as an in vitro model of acute inflammation, to test the ability of sera from adolescents with different MD adherence to tune the release of inflammatory cytokines. It has been reported that LPS-stimulated macrophages secrete pro-inflammatory cytokines, such as IL-6 and TNF-α, which indicate the presence of inflammation [44]. Interestingly, we observed that inflammatory macrophages cultured with serum from adolescents with optimal MD adherence display a reduced cytokine secretion with respect to that from poor and medium adherers, highlighting the potential anti-inflammatory properties exerted by sera from adolescents who had optimal compliance with MD pattern.

## 5. Conclusions

In conclusion, optimal adherence to the MD, rich in fruits, legumes, vegetables, and nuts, exerts anti-inflammatory and antioxidant properties in adolescents that may be due to the healthy quality of the MD pattern, which may have a positive impact on the prevention of metabolic and chronic diseases in adulthood.

## Figures and Tables

**Table 1:** General characteristics of the study population.

**Characteristics**	**Total Sample**	**Girls**	**Boys**
Subjects (number)	77	36	41
Age (years)	15.77 ± 1.07	15.72 ± 1.13	15.80 ± 1.03
Weight (Kg)	64.89 ± 12.59	58.55 ± 7.83	70.45 ± 13.4
Height (cm)	167.9 ± 8.21	161.54 ± 5.63	173.53 ± 5.6
BMI (Kg/m2)	22.87 ± 3.39	22.41 ± 2.93	23.26 ± 3.74
Overweight (%)	24.67	9.09	15.58
Obesity (%)	3.89	1.3	2.59
Waist (cm)	22.87 ± 3.39	22.41 ± 2.93	23.26 ± 3.74
Hip (cm)	73.23 ± 9.04	68.80 ± 6.38	77.10 ± 9.3
Waist/hip ratio	95.75 ± 8.05	95.08 ± 7.34	96.32 ± 8.67
BCM (%)	54.45 ± 3.73	53.38 ± 2.92	56.26 ± 3.43
FM (Kg)	14.37 ± 6.57	16.09 ± 5.55	12.85 ± 7.07
FFM (Kg)	50.78 ± 9.81	42.55 ± 3.47	58.02 ± 7.60
PhA (°)	6.34 ± 0.90	6.01 ± 0.93	6.63 ± 0.78
TBW (%)	58.09 ± 6.68	54.59 ± 5.54	61.17 ± 6.10
**General metabolic, Health, and Inflammatory Biomarkers**			
Glucose (mg/dL)	79.71 ± 6.76	77.77 ± 6.73	81.41 ± 6.39
Insulin (mU/L)	10.90 ± 7.81	9.50 ± 5.67	12.13 ± 9.18
HOMA-IR	2.16 ± 1.56	1.84 ± 1.1	2.39 ± 1.86
TG (mg/dL)	75.26 ± 61.94	70.67 ± 59.45	79.29 ± 64.50
Total Cholesterol (mg/dL)	150.83 ± 27.71	158 ± 28.35	144.54 ± 25.86
LDL (mg/dL)	85.51 ± 22.77	88.72 ± 25.65	82.68 ± 19.78
HDL (mg/dL)	50.21 ± 11.53	55.27 ± 11.89	45.76 ± 9.24
Creatinine (mg/dL)	0.89 ± 0.12	0.81 ± 0.09	0.96 ± 0.09
Urea nitrogen (mg/dL)	30.06 ± 6.48	28 ± 4.89	31.88 ± 7.18
Uric acid (mg/dL)	4.86 ± 1.40	4.26 ± 1.03	5.39 ± 1.42
Total bilirubin (mg/dL)	1.01 ± 0.56	0.84 ± 0.31	1.16 ± 0.69
Direct bilirubin (mg/dL)	027 ± 0.09	0.24 ± 0.06	0.30 ± 0.10
ESR (mm/h)	17.01 ± 11.01	23.28 ± 11.22	11.51 ± 7.35
CRP (mg/L)	1.20 ± 0.64	1.19 ± 0.60	1.21 ± 0.68
**Adherence to the MD**			
KIDMED Score (mean ± SD)	6.71 ± 2.58	6.5 ± 2.74	6.9 ± 2.46

BMI, body mass index; BCM, body cell mass; FM, fat mass; FFM, fat-free mass; PhA, phase angle; TBW, total body water; HOMA-IR, Homeostasis Model Assessment for estimating insulin resistance; TG, triglyceride; LDL, low density lipoprotein; HDL, high density lipoprotein; ESR, erythrocyte sedimentation rate; CRP, C-Reactive Protein.

**Table 2:** Total sample stratification with respect to the Mediterranean Diet adherence (KIDMED score).

KIDMED Score	Total Sample
Poor adherence (≤3)	7 (9%)
Medium adherence (4–7)	37 (48%)
Optimal adherence (≥8)	33 (43%)

**Table 3:** Energy and nutrients intake from 24 h recalls in total sample stratification with respect to the Mediterranean Diet adherence (KIDMED score).

	Adherence to the Mediterranean Diet			
Primary Energy Sources	Poor	Medium	Optimal	*p*-Value
Total Energy (kcal)	1437.85 ± 386.31	1716 ± 520.79	1707.42 ± 441.85	0.34 * 0.37 ¥ 0.10 §
Total Fat (g)	72.10 ± 19.62	79.23 ± 25.79	76.12 ± 19.62	0.70 * 0.93 ¥ 0.88 §
Total Carbohydrate (g)	137.15 ± 44.02	175.65 ± 76.12	169.26 ± 71.02	0.40 * 0.53 ¥ 0.93 §
Total protein (g)	55.45 ± 28.92	70.43 ± 26.65	81.31 ± 24.22	0.34 * 0.05 ¥ 0.19 §
Animal Protein (g)	31.30 ± 22.87	40.60 ± 27.21	49.99 ± 24.46	0.66 * 0.19 ¥ 0.28 §
Vegetable Protein (g)	18.23 ± 10.22	22.65 ± 12.18	23.80 ± 8.82	0.58 * 0.43 ¥ 0.89 §
**Fats**				
SFA (g)	38.86 ± 33.03	45.71 ± 20.15	48.18 ± 22.60	0.74 * 0.58 ¥ 0.89 §
MUFA (g)	39.65 ± 10.98	38.14 ± 16.25	52.07 ± 78.08	0.10 * 0.84 ¥ 0.51 §
PUFA (g)	35.61 ± 33.03	42.46 ± 20.15	44.93 ± 22.60	0.74 * 0.58 ¥ 0.89 §
Vegetable Fats (g)	47.66 ± 10.87	43.11 ± 17.81	43.54 ± 17.68	0.80 * 0.83 ¥ 0.99 §
Animal Fats (g)	14.97 ± 12.25	30.96 ± 24.01	26.06 ± 23.58	0.22 * 0.48 ¥ 0.65 §
Omega-3 Fatty Acids (g)	0.68 ± 0.23	1.16 ± 0.89	1.20 ± 0.74	0.32 * 0.27 ¥ 0.98 §
Omega-6 Fatty Acids (g)	6.69 ± 2.90	7.99 ± 5.29	7.57 ± 4.42	0.78 * 0.90 ¥ 0.93 §
EPA (g)	0.025 ± 0.04	0.14 ± 0.24	0.07 ± 0.14	0.30 * 0.70 ¥ 0.36 §
DHA (g)	0.02 ± 0.02	0.22 ± 0.69	0.14 ± 0.28	0.60 * 0.85 ¥ 0.75 §
PUFA:SFA ratio	0.82 ± 0.13	0.90 ± 0.07	0.90 ± 0.06	0.05 * **0.04 ¥** 0.96 §
Cholesterol (mg)	150.89 ± 114.63	233.55 ± 173.76	230.69 ± 175.99	0.47 * 0.50 ¥ 0.99 §
**Carbohydrates**				
Starch (g)	94.74 ± 37.64	104.73 ± 62.28	100.95 ± 51.90	0.90 * 0.96 ¥ 0.96 §
Soluble sugars (g)	21.97 ± 13.24	55.37 ± 33.23	55.019 ± 22.35	**0.01** * **0.01 ¥** 0.99 §
Glycemic Index	184.34 ± 330.02	59.75 ± 15.21	59.064 ± 16.17	**0.01** * **0.01 ¥** 0.99 §
Glycemic Load	70.92 ± 55.15	83.93 ± 54.39	82.64 ± 47.83	0.81 * 0.85 ¥ 0.99 §
**Fibers**				
Total dietary fiber	9.90 ± 4.90	13.32 ± 5.77	16.21 ± 5.37	0.30 * **0.02 ¥** 0.08 §
Soluble dietary fiber	0.97 ± 1.10	2.08 ± 1.25	2.64 ± 1.59	0.14 * **0.01 ¥** 0.23 §
Insoluble dietary fiber	3.27 ± 2.14	6.15 ± 4.68	8.043 ± 4.49	0.26 * **0.03 ¥** 0.18 §
**Vitamins**				
Vitamin A eq. Retinol	666.35 ± 563.48	922.60 ± 705.33	1147.81 ± 807.09	0.68 * 0.26 ¥ 0.42 §
Vitamin B1 (mg)	0.78 ± 0.30	1.52 ± 3.32	0.95 ± 0.27	0.72 * 0.98 ¥ 0.57 §
Vitamin B2 (mg)	0.79 ± 0.44	1.02 ± 0.40	1.35 ± 0.50	0.43 * **0.01 ¥ 0.01 §**
Vitamin B3 (mg)	13.37 ± 7.04	14.71 ± 8.24	18.74 ± 7.77	0.91 * 0.24 ¥ 0.09 §
Vitamin B5 (mg)	1.63 ± 1.16	2.33 ± 1.62	3.2 ± 1.87	0.59 * 0.07 ¥ 0.09 §
Vitamin B6 (mg)	1.35 ± 0.66	1.69 ± 1.35	1.98 ± 0.60	0.70 * 0.32 ¥ 0.50 §
Vitamin B8 (µg)	6.54 ± 5.77	12.78 ± 11.76	16.46 ± 11.68	0.38 * 0.09 ¥ 0.37 §
Folic Acid (µg)	165.46 ± 108.23	200.82 ± 100	263.29 ± 105.32	0.68 * 0.06 ¥ **0.04 §**
Vitamin B12 (µg)	1.45 ± 1.41	3.28 ± 2.70	3.90 ± 2.64	0.21 * 0.07 ¥ 0.59 §
Vitamin C (mg)	33.25 ± 37.75	84.58 ± 49.05	108.60 ± 72.64	0.10 * **0.01 ¥** 0.22 §
Vitamin K (µg)	0.00 ± 0.00	2.48 ± 5.14	3.86 ± 6.64	0.53 * 0.22 ¥ 0.56 §
Vitamin D (µg)	0.85 ± 0.58	2.87 ± 6.77	2.65 ± 3.87	0.63 * 0.70 ¥ 0.98 §
Vitamin E (mg)	10.69 ± 2.77	13.25 ± 6.77	12.79 ± 4.27	0.51 * 0.63 ¥ 0.94 §
**Total ORAC** (µmol TE)	1599.86 ± 1625.11	4196.97 ± 3069.62	6236.43 ± 5973.07	0.35 * **0.04 ¥** 0.16 §
**Minerals**				
Calcium (mg)	334.13 ± 243.55	543.93 ± 338.73	571.26 ± 266.03	0.62 * 0.91 ¥ 0.65 §
Phosphorus (mg)	657.19 ± 184.33	848 ± 426.36	986.47 ± 405.55	0.49 * 0.13 ¥ 0.34 §
Iodium (µg)	23.45 ± 19.43	80.36 ± 115.18	75.83 ± 66.92	0.29 * 0.36 ¥ 0.98 §
Sodium(mg)	1131.22 ± 1008.09	1319.69 ± 1137.96	1274.45 ± 1060.31	0.91 * 0.95 ¥ 0.98 §
Iron (mg)	5.98 ± 2.04	8.83 ± 5.47	9.22 ± 2.85	0.25 * 0.17 ¥ 0.92 §
Magnesium (mg)	118.26 ± 56.14	183.51 ± 120.74	217.01 ± 95.75	0.30 * 0.07 ¥ 0.39 §
Selenium (µg)	15.48 ± 9.47	35.6 ± 42.62	50.421 ± 48.61	0.51 * 0.14 ¥ 0.34 §
Potassium (mg)	1651.16 ± 829.74	2216.68 ± 866.57	2762.54 ± 787.02	0.23 * **0.01 ¥ 0.02 §**
Zinc (mg)	5.60 ± 1.40	8.79 ± 5.03	8.957 ± 3.43	0.16 * 0.14 ¥ 0.98 §
**Water** (g)	471.43 ± 292	767.21 ± 466.76	983.22 ± 507.56	0.29 * **0.03 ¥** 0.15 §

SFA, saturated fatty acid; MUFA, monounsaturated fatty acid; PUFA, polyunsaturated fatty acid; EPA, eicosapentaenoic acid; DHA, docosahexaenoic acid; ORAC, Oxygen Radical Absorbance Capacity; eq., equivalent. * Poor vs. Medium. ¥ Poor vs. Optimal. § Medium vs. Optimal. Statistical differences were evaluated by a one-way ANOVA test. In bold are reported statistically significant values.

## Data Availability

The data presented in this study are available in article and supplementary material.

## Supplementary Materials

The following are available online at https://www.mdpi.com/article/10.3390/antiox10081172/s1, Figure S1: Examples of photographs from the photo atlas showing portion sizes and the energy intake., Table S1: Anthropometric and body composition parameters, metabolic and health risk indicators in adolescents stratified with respect to the Mediterranean Diet adherence.
