# Supplementary material for: Potential Antioxidant and Anti-Inflammatory Properties of Serum from Healthy Adolescents with Optimal Mediterranean Diet Adherence: Findings from DIMENU Cross-Sectional Study
Source: Antioxidants (Basel). 2021 Jul 23;10(8):1172. doi: 10.3390/antiox10081172 (PMC8389034; doi:10.3390/antiox10081172)
Supplement: Supplementary file 1 [file antioxidants-10-01172-s001.zip › antioxidants-1305506-supplementary.pdf]

Figure S1: Examples of photographs from the photo atlas showing portion sizes and the energy intake.

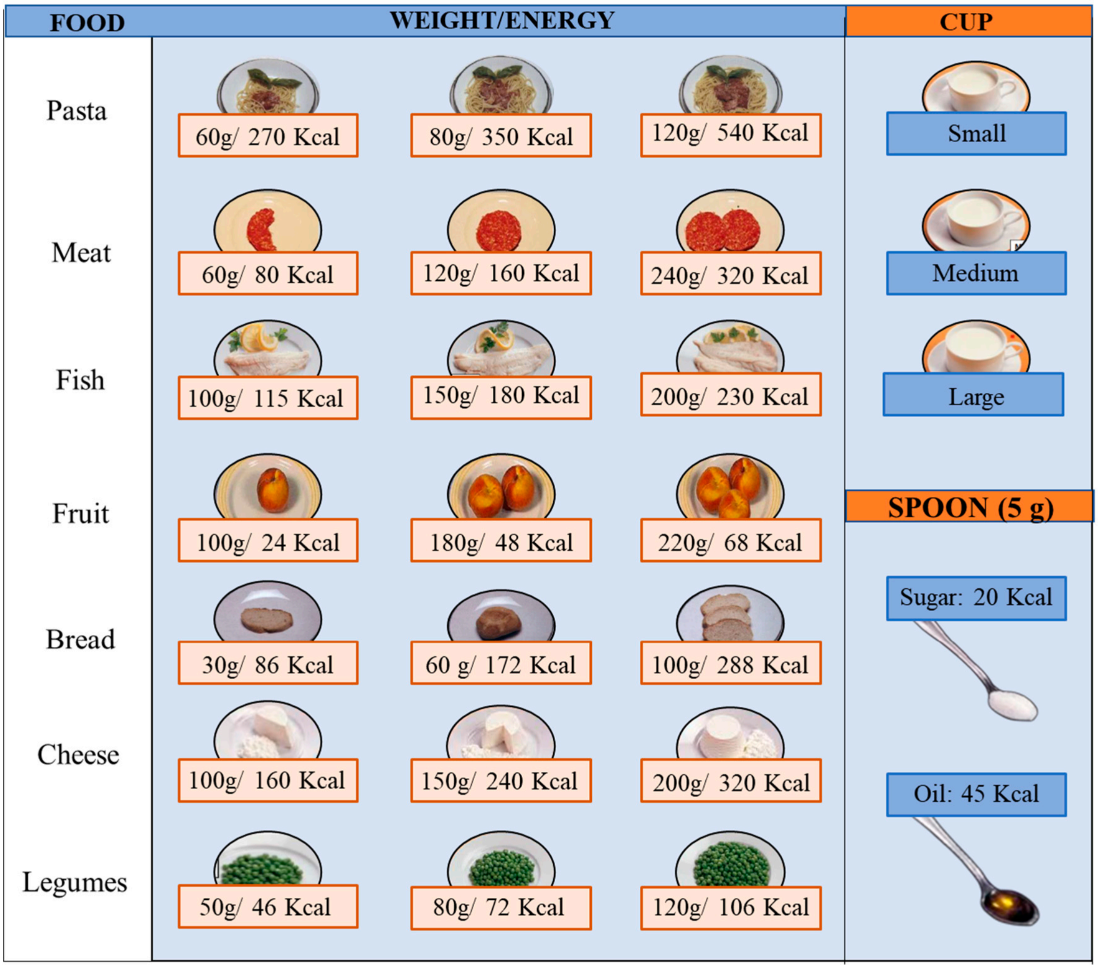

Table S1: Energy and nutrients intake from 24-hour recall in total sample.

**Primary energy sources**	**Total Sample**
Total Energy (kcal)	1687,04 ± 475,09
Total Fat (g)	77,25 ± 26,65
Total Carbohydrate (g)	169,42 ± 71,21
Total protein (g)	72,96 ± 28,27
Animal Protein (g)	43,78 ± 25,91
Vegetable Protein (g)	22,75 ± 10,79
**Fats**	
SFA (g)	20,01 ± 9,68
MUFA (g)	39,21 ± 15,44
PUFA (g)	8,87 ± 4,81
Vegetable Fats (g)	44,00 ± 16,83
Animal Fats (g)	26,66 ± 22,68
Omega-3 Fatty Acids (g)	1,14 ± 0,79
Omega-6 Fatty Acids (g)	7,39 ± 4,05
EPA (g)	0,09 ± 0,173
DHA (g)	0,16 ± 0,50
PUFA:SFA ratio	0,50 ± 0,29
Cholesterol (mg)	224,82 ± 169,00
**Carbohydrates**	
Starch (g)	169,42 ± 71,67
Soluble sugars (g)	52,18 ± 28,79
Glycemic Index	71,39 ± 20,18
Glycemic Load	83,72 ± 51,63
**Fibers**	
Total dietary fiber	14,25 ± 5,76
Soluble dietary fiber	2,22 ± 1,45
Insoluble dietary fiber	6,70 ± 4,58
**Vitamins**	
Vitamin A eq. Retinol	998,38 ± 738,32
Vitamin B1 (mg)	0,95 ± 0,85
Vitamin B2 (mg)	1,13 ± 0,49
Vitamin B3 (mg)	16,05 ± 7,96
Vitamin B5 (mg)	2,56 ± 1,72
Vitamin B6 (mg)	1,69 + 0,71
Vitamin B8 (μg)	13,82 + 11,43
Folic Acid (μg)	225,69 ± 105,94
Vitamin B12 (μg)	4,21 ± 8,43
Vitamin C (mg)	88,25 ± 62,73
Vitamin K (μg)	2,79 ± 5,51
Vitamin D (μg)	2,05 ± 2,89
Vitamin E (mg)	12,32 ± 3,66
**Total ORAC (μmol TE)**	1599,86 ± 1625,11
**Minerals**	
Calcium (mg)	534,45 ± 304,78
Phosphorus (mg)	657.19 ± 184.33

Iodium (μg)	64,93 ± 65,77
Sodium(mg)	1291,45 ± 1082,36
Iron (mg)	8,65 ± 4,24
Magnesium (mg)	191,69 ± 107,16
Selenium (μg)	37,39 ± 34,35
Potassium (mg)	2408,98 ± 852,20
Zinc (mg)	6.47 ± 0.08
**Water (g)**	660.71 ± 50.13

SFA, saturated fatty acid; MUFA, monounsaturated fatty acid; PUFA, polyunsaturated fatty acid; EPA, eicosapentaenoic acid; DHA, docosahexaenoic acid. ORAC, Oxygen Radical Absorbance Capacity.

Table S2: Anthropometric and body composition parameters, metabolic and health risk indicators in adolescents stratified with respect to the Mediterranean Diet adherence.

	Adherence to the Mediterranean Diet			
**Characteristics**	**Poor**	**Medium**	**Optimal**	**P-value**
Weight (Kg)	64.65±20.10	66.1±12.75	63.58±10.70	*0.96 ¥0.98 §0.69
Height (cm)	163.97±8.93	168.20±8.15	168.47±8.16	*0.43 ¥0.39 §0.99
BMI (Kg/m^2)	23.63±4.55	23.20±3.36	22.33±3.20	*0.95 ¥0.63 §0.53
Waist (cm)	72.93±12.30	73.42±9.14	73.08±8.47	*0.99 ¥0.99 §0.98
Hip (cm)	94.43±8.32	96.74±8.67	94.90±7.36	*0.77 ¥0.99 §0.61
Waist/hip ratio	0.77±0.07	0.76±0.06	0.77±0.06	*0.91 ¥1 §0.74
BCM (%)	54.63±3.66	54.13±3.92	54.77±3.61	*0.94 ¥0.99 §0.76
FM (Kg)	14.79±7.91	14.89±6.22	13.69±6.81	*0.99 ¥0.92 §0.73
FFM (Kg)	49.87±13.94	51.19±9.85	50.52±9.10	*0.94 ¥0.99 §0.96
PhA (°)	6.21±0.75	6.45±1.06	6.25±0.75	*0.81 ¥0.99 §0.65
TBW (%)	56.74±5.33	57.44±6.56	59.10±7.10	*0.96 ¥0.67 §0.55
**General metabolic, health and inflammatory biomarkers**				
Glucose (mg/dL)	80.14±5.73	80.10±7.40	79.18±6.35	*1 ¥0.94 §0.84
Insulin (mU/L)	11.64±11.97	12.11±7.96	9.38±6.51	*0.99 ¥0.77 §0.31
HOMA-IR	2.30±2.41	2.43±1.60	1.83±1.26	*0.98 ¥0.74 §0.24
TG (mg/dL)	78.86±71.09	82.49±79.80	66.39±29.27	*0.52 ¥0.88 §0.50
Total Cholesterol (mg/dL)	143.71±18.43	150.84±29.75	152.33±27.41	*0.81 ¥0.74 §0.97
LDL (mg/dL)	75.86±17.68	85.92±22.06	87.09±24.55	*0.54 ¥0.47 §0.97
HDL (mg/dL)	50.86±18.13	48.51±8.93	51.97±12.55	*0.87 ¥0.97 §0.43
Creatinine (mg/dL)	0.92±0.13	0.90±0.12	0.88±0.12	*0.93 ¥0.78 §0.85
Urea nitrogen (mg/dL)	29.28±4.54	29.05±7.13	31.36±5.96	*0.99 ¥0.72 §0.30
Uric acid (mg/dL)	4.49±0.83	4.71±1.21	4.99±1.46	*0.91 ¥0.62 §0.64
Total bilirubin (mg/dL)	1.02±0.44	0.90±0.42	1.14±0.70	*0.86 ¥0.87 §0.18
Direct bilirubin (mg/dL)	0.29±0.09	0.26±0.09	0.28±0.09	*0.71 ¥ 0.94 §0.72
ESR (mm/h)	15.71±5.85	17.51±12.04	16.72±10.85	*0.99 ¥0.96 §0.75
CRP (mg/L)	1.25±0.67	1.30±0.83	1.08±0.31	*0.98 ¥0.80 §0.33

* Poor vs Medium. ¥ Poor vs Optimal. § Medium vs Optimal. Statistical differences were evaluated by one-way ANOVA test.
